# Does Practice Match Protocol? A Comparison of Triage-to-Provider Time Among More vs. Less Acute Emergency Department Patients

**DOI:** 10.7759/cureus.66079

**Published:** 2024-08-03

**Authors:** Temesgen T Tsige, Rida Nasir, Daisy Puca, Kevin Charles, Sandhya LoGalbo, Lisa O Iyeke, Lindsay Jordan, Melva O Morales Sierra, David Silver, Mark Richman

**Affiliations:** 1 Department of Emergency Medicine, Long Island Jewish Medical Center, Northwell Health, Long Island, USA; 2 Department of Emergency Medicine, Glen Cove Hospital, Northwell Health, Glen Cove, USA

**Keywords:** healthcare quality improvement, emergency medicine, emergency severity index (esi), triage, split-flow model

## Abstract

Introduction

The Emergency Severity Index (ESI) stratifies emergency department (ED) patients for triage, from “most acute” (level 1) to “least acute” (level 5). Many EDs have a split flow model where less acute (ESI 4 and 5) are seen in a fast track, while more acute (ESI 1, 2, and 3) are seen in the acute care area. A core principle of emergency medicine (EM) is to attend to more acute patients first. Deliberately designating an area for less acute patients to be initially assessed quickly by a first provider might result in them being seen before more acute patients. This study aims to determine the percentage of less acute patients seen by a provider sooner after triage than more acute patients who arrived within 10 minutes of one another. Additionally, this study compares the fast track and acute care areas to see if location affects triage-to-provider time.

Methods

A random convenience sample of 252 ED patients aged ≥18 was taken. Patients were included if their ESI was available for the provider during sign-up. Patients were excluded if they were directly sent to the ED psychiatric area or attended to by the author. We collected data on the ESI level, time stamps for triage and first provider sign-up, and the location to which the patient was triaged (fast track vs. acute care). Paired patients’ ESI levels, locations, and triage and first provider sign-up times were compared.

Results

The study included 126 pairs of patients. There was a statistically significant difference in triage-to-provider times for paired ESI 2 vs. 3 patients (60.5 vs. 35.5 minutes, p = 0.0007) and overall paired high- vs. low-acuity patients (55 vs. 39.5 minutes, p = 0.004). However, in 34.8% of paired ESI 2 vs. 3 patients, the ESI 3 patient was seen prior to the paired ESI 2 patient, and in 39.4% of overall paired high vs. low acuity patients, the less acute patient was seen before the more acute patient. Additionally, patients in the acute care area had significantly shorter median triage-to-provider times (~40 minutes) compared to those in the fast track area for ESI 2 (acute care) vs. ESI 3 (fast track) and overall high acuity (acute care) vs. low acuity (fast track). Nonetheless, approximately one-third of ESI 3 patients triaged to fast track were seen before ESI 2 patients triaged to the acute care area.

Conclusion

The split flow model reduces overall ED length of stay, improving flow volume, revenue, and patient satisfaction. However, it comes at the expense of the fundamental ethos of EM and potentially subverts the intended triage process. Although most more acute patients are seen by a provider sooner after triage than less acute patients, a substantial number are seen later, which could delay urgent medical needs and negatively impact patients’ outcomes. Furthermore, patients triaged to acute care are, in general, seen sooner post-triage than identical-ESI-level fast track patients, suggesting fast track might not function as intended (for low-acuity patients to be quickly assessed and initiate diagnostic and treatment plans). We intend to follow this exploratory study with a more comprehensive, multivariate analysis that will consider confounding variables such as initial vital signs, how busy a provider was that day, etc. The future study will also examine patient outcomes to determine the impact on more acute patients of the split flow model and, in particular, on less acute patients being seen sooner by a first provider.

## Introduction

The Emergency Severity Index (ESI) is a standard means of stratifying emergency department (ED) patients for triage so as to be seen in a particular order (from “most acute” to “least acute”) [1]. ESI levels range from 1 (most acute) to 5 (least acute). ESI levels 1 and 2 are “emergent” (require immediate assessment and intervention); ESI level 3 is “urgent” (can safely wait a short amount of time); and ESI levels 4 and 5 are “non-urgent” (can safely wait a long time) [2].

The original basis for determining an ESI level was a combination of acuity and the expected number of resources the patient would use in the ED. In practice, an ESI level is assigned based on the gestalt and experience of the person performing triage, supplemented with standardized criteria such as vital sign abnormalities [1].

The ethics and imperatives of emergency medicine (EM) require providers to attend to the sickest patients first [3]. It should, therefore, not routinely occur that less acute patients (ESI level 4 or 5) should be seen sooner after arrival than more acute patients (ESI levels 1, 2, or 3) who arrive at approximately the same time.

Many EDs have instituted a split flow model [4] in which potentially less acute patients (ESI 4 and 5) are seen on a “fast track” (where patients have a lower expected length of stay ((LOS)) while more acute patients (ESI 1, 2, and 3) are seen in a separate area. There are several variations in the split flow model. In one variation, a licensed independent provider serves with, or even in lieu of, the triage nurse and, after a brief history and physical examination, orders studies or may discharge the patient [5]. In another variant, a nurse triages a patient to either a low-acuity (e.g., fast track) area or an acute care area [6]. In this manner, lower-acuity patients can be seen rapidly, which is beneficial to both the patient and the ED, although it should be noted that many variables affect “triage-to-doctor time” (e.g., physician vs. mid-level provider staffing mix; physician-to-patient ratio).

Split flow models have been associated with decreased LOS for low acuity and for overall ED patients [4]. This can generate substantial revenue from the volume and difference between billing collections and resources expended on low-acuity patients, especially because many EDs charge a facility fee [7] to each patient, regardless of the level of complexity.

Wait times to be seen by a provider increase with increasing ED volume [8]. Consequently, there are frequent circumstances where high-acuity (particularly ESI 2) patients have prolonged wait times [9]. The presence of the split flow model introduces the possibility that a more acute patient (e.g., ESI 2) triaged to an acute care area will be seen later after arrival than a less acute patient (ESI 3, 4, or 5) triaged to a fast track area. Such a situation would occur because, rather than a provider being redeployed from the fast track to see the more acute patient quickly, that provider is retained in the fast track area to continue seeing lower-acuity patients.

This study was an exploratory, univariate analysis to determine what proportion of less acute patients have lower triage-to-provider time than more acute patients and quantify the difference in triage-to-provider time between more vs. less acute patients. If, indeed, a substantial proportion of less acute patients are seen prior to more acute patients, we will follow with multivariate analysis, considering confounding variables such as initial vital signs, the number of patients seen by the provider that shift (a proxy variable for how busy the provider was that day), etc.

This article was previously posted to the medRxiv preprint server on February 13, 2024.

## Materials and methods

The Long Island Jewish Medical Center (LIJMC) is a 583-bed tertiary-care academic hospital serving a racially and socioeconomically diverse population. The adult ED sees approximately 100,000 patients per year and has an internal area designated for psychiatric patients. Nurses triage patients who arrive either by ambulance or who “walk in” to either the psychiatry area, a 20-bed fast track area (for ESI levels 3, 4, and 5 patients), or the acute care area (to which all ESI levels can be sent). As part of the triage process, a brief chief complaint and vital signs are obtained, as well as blood sugar measurement and ECG evaluations, as indicated. The ED does not utilize a “provider-up-front” mode whereby a provider assists with the triage process or rapidly screens a patient and orders studies or medications. The fast track has one room with equipment for ophthalmologic examinations and one room with a bed and equipment for gynecologic examinations; the acute care area does not have such designated rooms. While the area to which most ESI 1, 2, 4, and 5 patients should be triaged is usually clear (ESI 1 and 2 to acute care, ESI 4 and 5 to fast track), this is less clear among the ESI 3 group, which comprises patients with substantial heterogeneity in potential severity (e.g., older patients with chest pain; young men with right lower quadrant pain). Consequently, the guidelines regarding the area to which ESI 3 patients are triaged are flexible and based on estimated resources needed, professional judgment, and bed availability in various ED areas. If the fast track area is overwhelmed with patients, then ESI 4 and 5 patients may be sent to the acute care area. Approximately 55% of ED volume is triaged in the fast track area, 35% in the acute care area, and 10% in the psychiatric area.

All ED treatment areas are staffed by patient care assistants (similar to medical assistants), ED technicians (repurposed paramedics), nurses, and providers (attending and resident physicians, physician assistants, and (rarely) nurse practitioner mid-level providers). Throughout the ED, laboratory or radiology studies are ordered only by providers after they have seen a patient and not by staff other than providers (e.g., by a nurse operating under standardized procedure).

We performed an exploratory, univariate analysis to determine what percentage of lower-acuity patients are seen before higher-acuity patients and whether this varies according to the ED location to which the patients are triaged. A convenience sample of 126 pairs of patients aged ≥18 years was taken between April 24 and December 13, 2023. Patients arriving during any shift when a physician author (MR and DS) was present were included. Patient data was captured by research assistants during a wide variety of possible sampling times, given the variability in ED shiftwork (any time of day, evening, or night; weekdays and weeknights). Any pair of patients whose triage times were within 10 minutes of each other were selected and included as long as the provider sign-up time for each patient was after triage, so the ESI level was available for the provider to see, as that might affect how the provider prioritized which patient to see first. The patients in a pairing could have been triaged either in the same area (e.g., both patients to fast track) or in different areas (e.g., one patient to acute care, one patient to fast track). Patients were excluded if they were either directly sent to the psychiatric area of the ED after triage or if any of the authors was their attending physician.

We conducted a real-time review of the electronic health record (EHR) time stamps of patients arriving in the ED, looking for patients with different ESI levels and with triage time stamps within 10 minutes of each other. Patients were selected after the attending physician signed up for them so that it was known which patients either physician author (MR or DS) had signed up for; such patients were excluded from the study. Patients with ESI 1 or 2 were considered “high acuity;” those with ESI 3, 4, and 5 were considered “low acuity.” Variables collected were ESI level, ED location to which the patient was triaged (fast track vs. acute care area), and time stamps for triage time and time of first provider sign-up. Attending and resident physicians, PAs, and NPs were considered to be providers.

Two main types of analyses were performed. First, triage-to-provider time was treated as a continuous variable; the median triage-to-provider time of all patients with particular ESI levels was compared (e.g., the median triage-to-provider time of all patients with ESI 2 vs. all patients with ESI 3). Second, triage-to-provider time was treated as a binary variable: within a paired group (e.g., all ESI 1 vs. 3 pairs, all ESI 2 vs. 4 pairs), we calculated the percentage of cases in which the less acute patient was seen before or after the more acute patient (e.g., among all pairs of ESI 1 vs. 3 patients, in what percent was the ESI 3 patient seen prior to or subsequent to the ESI 1 patient). The time stamp “triage” (rather than “arrival time” or “room time”) was chosen because triage is the first time during an ED visit that a licensed healthcare provider has determined acuity and can expedite care for more acute patients. We also investigated the influence of triage destinations (fast track vs. acute care area) on triage-to-provider time for different ESI levels.

Descriptive statistics were used to describe the percentage of patients of each ESI level triaged to the fast track vs. acute care areas. Median times from triage to provider sign-up were determined, and median time differences were calculated. The Mann-Whitney test was used to compare median times. Statistical significance was set a priori at p < 0.05.

This study was deemed exempt by the Institutional Review Board (IRB #23-0169).

## Results

A total of 126 pairs of patients (252 patients overall) with different ESI levels whose triage time was within 10 minutes of each other were included. A similar proportion of more acute and less acute patients arrived first in the pairing (p = 0.72) (Table 1). Forty percent of patients were male, and one-third were ≥65 years old. Nearly half (44%; 111/252) of patients were ESI 3. No patients were triaged at an ESI level of 5.

**Table 1:** Percentage of times the more acute patient in the pair arrived before the less acute patient

Acuity	Number of patients who arrived first	Percentage of patients who arrived first	p-value
Less acute	65	51.6	0.72
More acute	61	48.4	

More than 74% of the patients assigned fast track were ESI 3, compared to only 22% in the acute care area. Similarly, in the acute care area, 70% of the patients were assigned ESI 2, compared to 5% in the fast track (Table 2).

**Table 2:** ESI level assigned, fast track vs. acute care area ED, emergency department; ESI, Emergency Severity Index

ESI level assigned	Percentage of all patients assigned this ESI level	Percentage of acute care ED patients with this ESI level	Percentage of fast track patients with this ESI level
1	1.6	3	0
2	42.1	70	5
3	44.4	22	74
4	11.9	5	21

Median triage-to-provider time among more acute patients triaged to the acute care ED was approximately 40 minutes less than for less acute patients triaged to fast track (76 vs. 33 minutes, p = 0.0008, for ESI 2 vs. 3; 71.5 vs. 33.5 minutes, p = 0.0004 for overall more vs. less acute) (Table 3).

**Table 3:** Triage-to-provider time comparisons for different pairs of ESI levels triaged to acute care vs. fast track Location is placed in parentheses; for instance, 2 (acute care) vs. 3 (fast track) indicates that ESI 2 patients were treated in acute care compared to ESI 3 patients treated in the fast track area. ESI, Emergency Severity Index

ESI comparison group	ESI level	The median time between triage to provider (minutes) (N)	p-value
ESI 1 in acute care vs. ESI 3 in fast track	1	19.5 (2)	0.33
	3	80.5 (2)	
ESI 2 in acute care vs. ESI 3 in fast track	2	33 (63)	0.0008
	3	76 (63)	
ESI 2 in acute care vs. ESI 4 in fast track	2	36.5 (8)	0.64
	4	42 (8)	
ESI 3 in acute care vs. ESI 4 in fast track	3	42 (3)	1
	4	29 (3)	
Acute care vs. fast track	Acute care	33.5 (76)	0.0004
	Fast track	71.5 (76)	

There was a statistically significant difference in triage-to-provider times for paired ESI 2 vs. 3 patients (60.5 vs. 35.5 minutes, p < 0.001) and overall paired high- vs. low-acuity patients (55 vs. 39.5 minutes, p = 0.004) (Table 3). However, in 34.8% of paired ESI 2 vs. 3 patients, the ESI 3 patient was seen prior to the paired ESI 2 patient, and in 39.4% of overall paired high vs. low acuity patients, the less acute patient was seen before the more acute patient (Table 4).

**Table 4:** Triage-to-provider time comparisons for different pairs of ESI levels ESI, Emergency Severity Index

ESI comparison group	ESI level	Median triage-to-provider time (minutes) (N)	p-value
1 vs. 3	1	19.5 (4)	0.2
	3	62 (4)	
2 vs. 3	2	35.5 (92)	0.0007
	3	60.5 (92)	
2 vs. 4	2	39 (15)	0.69
	4	27 (15)	
3 vs. 4	3	63 (15)	0.4
	4	41 (15)	
More acute vs. less acute	More acute	39.5 (126)	0.004
	Less acute	55 (126)	

An even higher percentage of less acute patients were seen first among ESI 2 vs. 4 and 3 vs. 4 patients (53.3% and 66.7%, respectively) (Table 5). However, perhaps owing to the small sample sizes of these pairings, these differences did not reach statistical significance.

**Table 5:** Percentage of time the less acute patient was seen before the more acute patient in the pair ESI, Emergency Severity Index

ESI comparison group	Comparison group (N)	Percentage of pairs in which less acute patients are seen first (N)	Median time difference (minutes)
1 vs. 3	4	0 (0)	30
2 vs. 3	92	34.8 (32)	13
2 vs. 4	15	53.3 (8)	-2
3 vs. 4	15	66.7 (10)	-11
More acute vs. less acute	126	39.4 (50)	9

Nonetheless, approximately one-third of ESI 3 patients triaged to fast track were seen before ESI 2 patients triaged to the acute care area (Table 6).

**Table 6:** Percentage of fast track patients with shorter triage-to-provider time compared with acute care area patients for various ESI level groupings ESI, Emergency Severity Index

Comparison group	Total comparison	Percentage of fast track patients seen first (N)	Median time difference (minutes)
ESI 1 (acute care) vs. ESI 3 (fast track)	2	0 (0)	61
ESI 2 (acute care) vs. ESI 3 (fast track)	63	31.75 (20)	23
ESI 2 (acute care) vs. ESI 4 (fast track)	8	37.5 (3)	12
ESI 3 (acute care) vs. ESI 4 (fast track)	3	33.3 (1)	3
Overall acute care vs. fast track	76	31.6 (24)	19.5

Among ESI level 3 patients generally (not paired, because this study looked at pairs of different ESI levels), patients triaged to the acute care area had a median triage-to-provider time 30 minutes sooner than those triaged to the fast track (p = 0.008) (Table 7).

**Table 7:** ESI 3 triage-to-provider times for fast track vs. acute care area ESI, Emergency Severity Index

Location	Median triage-to-provider time (minutes)	p-value
Acute care area	46	0.008
Fast track	76	

## Discussion

The split flow model of ED care has been associated with decreased overall LOS, both among patients triaged to the low-acuity area and those triaged to the higher-acuity areas. This is done to increase overall ED flow volume, revenue, and patient satisfaction. The split flow model adopted by the LIJ ED involves nurses triaging patients to either an acute care area or a fast track area. In contrast to other split flow models, in which there is a provider in triage, the LIJ model requires patients to still wait for a provider after they have arrived at either acute care or fast track areas.

In EM, there is a tension between an ED’s financial and patient satisfaction goals to see less acute patients quickly vs. the ethical responsibility to see more acute patients quickly [10,11]. This study reveals the unintended consequences of LIJ’s split flow model. Namely, it comes at the expense of the fundamental ethos/tenet of EM and subverts the intended triage process in such a way that a substantial number (~40% in this study) of patients deemed by the ESI triage level to be of lower acuity are seen before patients triaged as having higher acuity.

Not surprisingly, the study found that more acute patients were seen significantly faster than less acute patients in two different comparison groups: ESI 2 vs. 3 (the most common comparison pair) and overall high- vs. low-acuity groups. However, even within these groups, a large percentage of less acute patients (34.8% and 39.4%, respectively) were seen before more acute patients. Similar findings were also observed in other comparison groups, but not at statistically significant levels.

While the overall trend favors more acute patients being seen sooner after triage, the high percentage of less acute patients seen before more acute patients is potentially harmful, as it could delay care for those with urgent medical needs and negatively impact their outcomes. Such occurrences were noted by Stanford University operational researchers in a study at San Mateo Medical Center, which noted low-acuity patients increase wait times for high-acuity patients through delays in arrival-to-provider time [12].

Additionally, patients in the acute care area were seen significantly sooner post-triage than those in the fast track area in two comparison groups: ESI 3 (fast track) vs. ESI 2 (acute care) and overall less acute (fast track) vs. more acute (acute care). This finding raises questions about how effective the split flow model, particularly the fast track area, is in achieving its intended goal of allowing less acute (less sick) patients to be seen by a provider faster. Even among ESI level 3 patients only, patients triaged to fast track were seen 28 minutes later than those triaged to the acute care ED (p = 0.008). This finding further strengthens the evidence that the fast track is not functioning as a “fast track.” The heterogeneous/diverse clinical profiles among ESI 3 patients may contribute to their longer wait times to be seen in the fast track area [13].

Although the fast track is intended to see ESI levels 3, 4, and 5, five ESI 2 patients in our data set were triaged to fast track. A deeper investigation into these cases showed that one patient’s chief complaint was concern for a corneal ulcer; two were related to gynecology (concern for ectopic pregnancy; hyperemesis gravidarum); and two were employees with bloodborne pathogen exposures. These five patients were likely sent to fast track because fast track has a room with special equipment for ophthalmologic examinations and a room with a special bed and equipment for gynecologic examinations. In addition, employees with bloodborne pathogen exposures are triaged as an ESI 2 (even when not very ill) and sent to fast track so they can be seen quickly, both for purposes of providing them post-exposure HIV prophylaxis and to allow them to return to their job quickly.

While the ED should try, as often as possible, to see more acute patients sooner from triage than less acute patients, it would not be reasonable to suggest every more acute patient be seen sooner. This is because, while less sick patients are waiting to be seen, more sick patients will likely arrive, thereby relegating less sick patients to the “back of the line” over and over. Several options might relieve this conflict between the desire to see lower-acuity patients quickly and the imperative to see higher-acuity patients quickly: (1) Institutions should consider redistributing their workforce such that providers are active in the triage process (i.e., greatly minimize the time from “arrival to the provider”). (2) EDs can eliminate the split flow model and incorporate both less acute and more acute patients in the same areas of the ED, so providers can see a mix of both levels of patients. (3) Organizations can determine that less sick patients should be expected to wait a reasonable period of time (perhaps two hours) before being seen. Once they reach the two-hour limit, a provider could be notified to see them expeditiously, unless they are actively occupied with a more sick patient (i.e., not just waiting for the next more sick patient to be brought to their treatment area). (4) Finally, under the Emergency Medical Treatment & Labor Act, EDs are required to provide patients with a medical screening examination (MSE) to assess their level of sickness [14]. While the MSE can be performed by medical personnel qualified for the task rather than a licensed independent provider, in practice, it is typically conducted by such a provider. This MSE often evolves into a full assessment and treatment visit, even for minor complaints [15]. In New York, such providers could rapidly discharge low-acuity patients, either to home or to rapid follow-up at a facility for lower-acuity visits (e.g., urgent care centers), including one owned and operated by the same corporation that owns/operates the ED [15].

This study has several limitations. First, it was a single-center study; results may differ at other institutions, particularly because LIJ ED’s triage patterns may not reflect those in other EDs. A very high percentage (74%) of LIJ’s fast track patients are ESI 3, and approximately 55% of the ED volume comes through fast track, for a total of ~41,000 fast track ESI 3 patients/year. Other EDs may reserve their fast track areas primarily for ESI 4 and 5 patients. Second, we used the time stamp of provider sign-up to indicate when a provider first saw the patient. However, sometimes a provider might sign up for a patient shortly after they are triaged to that provider’s area, without seeing the patient until the patient arrives in the provider’s area. This might occur because, after the triage process, triage nurses often indicate in the ED EHR’s “Status” bar the ED area to which the patient will be sent. The provider might then sign up for the patient once they become aware the patient has been triaged to their area, without actually seeing the patient until the patient arrives in that area. In such cases, there will be a delay between when the provider signs up for the patient and when the provider actually sees the patient; the time stamp “Provider sign-up,” therefore, will not accurately reflect when the patient was seen. In such circumstances, signing up for the patient indicates not the “time seen,” but, rather, the time the provider took responsibility for the patient. Third, this being an exploratory study, some comparison groups had small sample sizes, while others were relatively large (44% (111/252) of patients were ESI 3). This uneven distribution may have limited the ability to detect whether differences truly existed. For example, in Table 2, the comparison of between-group median “triage-to-provider time” subgroups such as "ESI 1 vs. ESI 3" had very few subjects. The lack of significant differences in median “triage-to-provider time” may be due to the small number of subjects rather than to a true lack of difference. Finally, this study did not look at outcomes. The next step in our research will be to examine the outcomes (total ED LOS, disposition (e.g., admit vs. discharge), and 30-day return visits) of patients in our study.

## Conclusions

In our ED, the split flow model (which is partly contingent on the ESI triage process) most of the time results in more acute patients being seen sooner after triage than less acute patients, which is consistent with the ethos of EM. However, a substantial percentage (~40%) of less acute patients are still seen sooner post-triage than more cute patients. This phenomenon is worthy of additional studies, including a multivariate analysis to determine whether confounding variables may account for this observation, as well as studies at different institutions and follow-up patients to assess whether seeing less acute patients prior to more acute ones is associated with adverse outcomes.
